# 

**Published:** 1845-03

**Authors:** 


TERMS, ONE DOLLAR A YEAR, IN ADVANCE.

### THE ILLINOIS MEDICAL & SURGICAL JOURNAL.

EDITED BY

JAMES V. Z. BLANEY, M.D.

**Professor, &c.**

CHICAGO, ILL.

PUBLISHED BY ELLIS & FERGUS,

BOOK AND JOB PRINTERS,

SALOON BUILDINGS, CLARKE STREET,

1845.

